# Splenic Artery Pseudoaneurysm Presenting as Massive Hematemesis: A Diagnostic Dilemma

**DOI:** 10.1155/2014/501937

**Published:** 2014-02-13

**Authors:** Peeyush Varshney, Bhupen Songra, Shivank Mathur, Sudarshan Gothwal, Puneet Malik, Mahnedra Rathi, Rajveer Arya

**Affiliations:** General Surgery, SMS Medical College, B-207 Janta Colony, Jaipur, Rajasthan 302004, India

## Abstract

*Introduction*. Splenic artery Pseudoaneurysm, a complication of chronic pancreatitis, presenting as massive hematemesis is a rare presentation. *Case Report*. We present a case of 38-year-old male admitted with chief complaints of pain in the upper abdomen and massive hematemesis for the last 15 days. On examination there was severe pallor. On investigating the patient, Hb was 4.0 gm/dL, upper GI endoscopy revealed a leiomyoma in fundus of stomach, and EUS Doppler also supported the UGI findings. On further investigation of the patient, CECT of the abdomen revealed a possibility of distal pancreatic carcinoma encasing splenic vessels and infiltrating the adjacent structure. FNA taken at the time of EUS was consistent with inflammatory pathology. Triple phase CT of the abdomen revealed a splenic artery pseudoaneurysm with multiple splenic infarcts. After resuscitation we planned an emergency laparotomy; splenic artery pseudoaneurysm densely adherent to adjacent structures and associated with distal pancreatic necrosis was found. We performed splenectomy with repair of the defect in the stomach wall and necrosectomy. Postoperative course was uneventful and patient was discharged on day 8. *Conclusion*. Pseudoaneurysm can be at times a very difficult situation to manage; options available are either catheter embolisation if patient is vitally stable, or otherwise, exploration.

## 1. Introduction

Splenic artery pseudoaneurysm is a rare complication of pancreatitis often presenting as fatal complications such as rupture and bleeding. Proper history and physical examination and systemic approach to investigation are often needed to confirm diagnosis. Splenic artery pseudoaneurysm presents in a very confusing manner and symptoms are often nonspecific and require high index of suspicion for diagnosis. Due to life threatening complication prompt diagnosis is the key for successful outcome.

## 2. Case Report

**Figure 1:**
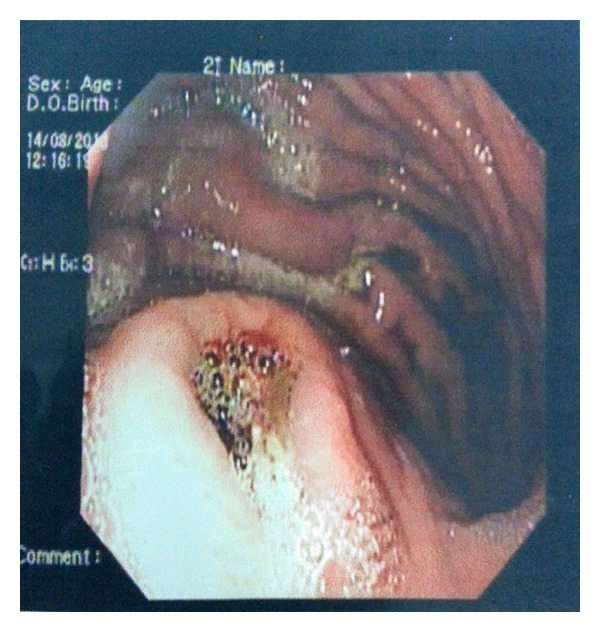
UGI endoscopy showing a picture of leiomyoma.

**Figure 2:**
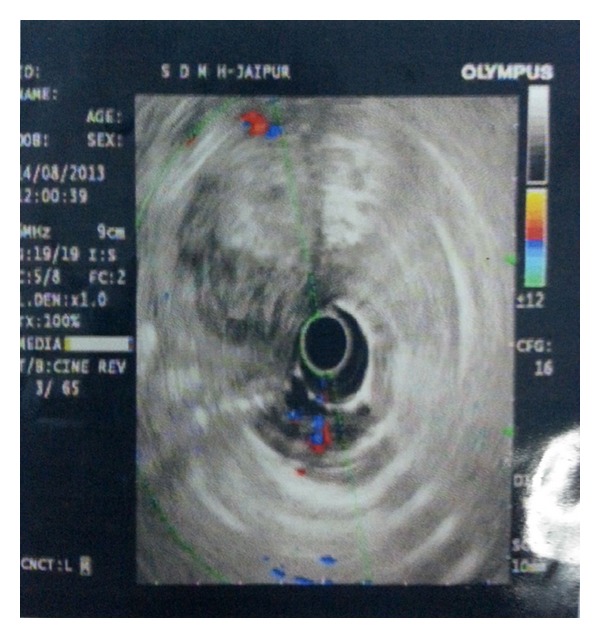
EUS Doppler depicting a mass in wall of stomach.

**Figure 3:**
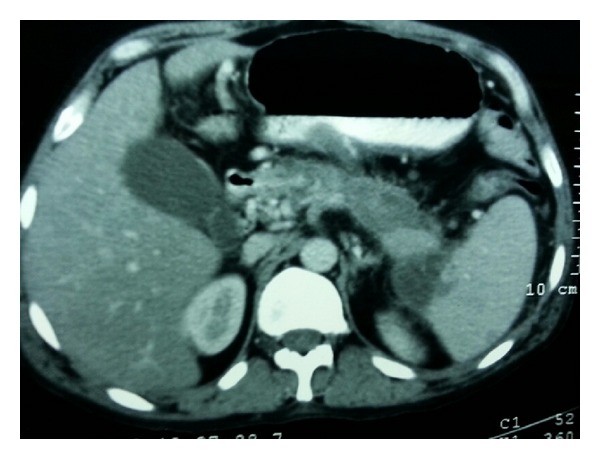
CT scan of the abdomen showing a pancreatic tail mass.

**Figure 4:**
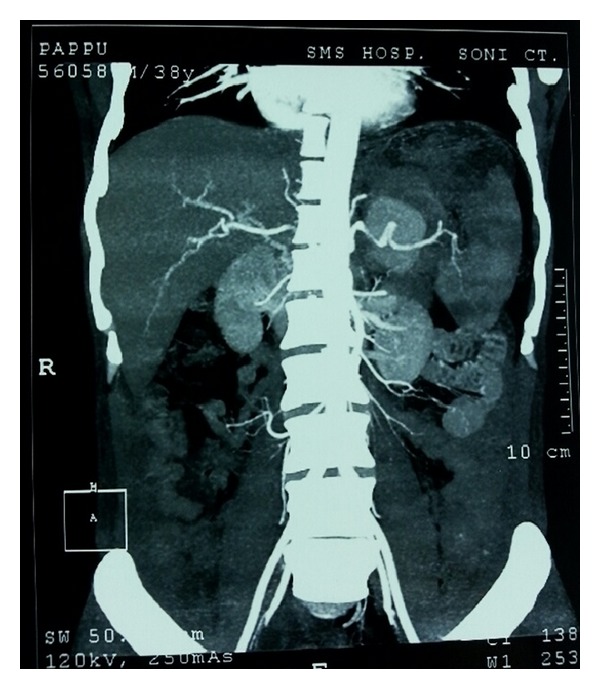
Triple phase CT finally giving the diagnosis of pseudoaneurysm.

**Figure 5:**
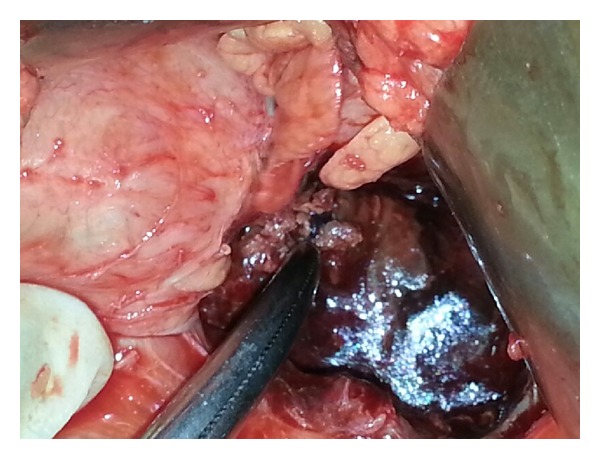
Per operative photograph of the patient showing lesser sac with pseudoaneurysm and necrosed part of pancreas.

A 38-year-old male was admitted to our tertiary hospital with chief complaints of pain in upper abdomen and massive hematemesis for the last 15 days. Patient had 4-5 episodes of vomiting which was red in color and 200 mL at a time. He also had mild dull aching pain and malena for the last 2 weeks. Patient was a chronic alcoholic. On examination there was severe pallor. On investigating the patient, Hb was 4.0 gm/dL, and other blood investigations were within normal limits. He already had an upper GI endoscopy (Figure 1) done outside stating large ulcer with undermined edge along lesser curvature at fundus with no active bleed. EUS Doppler done outside also (Figure 2) suggested a possibility of a heterogeneous mass lesion in fundus, likely leiomyoma. FNA which was taken at that time showed inflammatory cells. Patient also had a CT report which stated (Figure 3) a possibility of distal pancreatic carcinoma encasing splenic vessels and infiltrating the adjacent structure. After referring to gastroenterologist at our institute, repeat UGIE suggested a large submucosal lesion of 2.5 × 2.5 cm with central necrotic ulcer with evidence of recent bleeding (leiomyoma). As there was contradiction in findings of UGIE and CT, we proceeded in direction having probable diagnosis of some pancreatic neoplasm invading the wall of stomach. But as the stomach in CECT was normal and patient was bleeding recurrently we decided to do a triple phase CT before concluding our diagnosis. We finally got a triple phase CT of the abdomen (Figure 4) done at our institute which revealed a splenic artery pseudoaneurysm with multiple splenic infarcts. After resuscitation and transfusing 5 units of PRC we planned a laparotomy. On exploration splenic artery pseudoaneurysm densely adherent to adjacent structures and associated with distal pancreatic necrosis was found (Figure 5). On inspecting the stomach there was a small (0.5 × 0.5 cm) defect (hole/perforation) in the posterior wall with surrounding 0.5 cm area of hyperemia. We performed a splenectomy with proximal ligation of feeding vessel with necrosectomy. The stomach was primarily repaired with omental strengthening. Postoperative course was uneventful and patient was discharged on day 8. EUS done on postoperative day 3 was also normal with no evidence of bleeding. We finally concluded that proximal feeding splenic vessel might be a cause of hemorrhage as patient fared satisfactorily after surgery.

## 3. Discussion

There are various causes of splenic artery pseudoaneurysm such as pancreatitis 52%, abdominal trauma 29%, postoperative complication 3%, and peptic ulcer disease 2% [1]. Due to the enzymatic digestion of splenic and pancreatic arteries there is weakening of vessel wall which may sometimes erode the adjacent organ [2]. Peculiar feature differentiating splenic artery aneurysm from splenic artery pseudoaneurysm is that the latter is always symptomatic [3]. The most common presentation is upper or lower GI bleed (26.2%), hemorrhage into pancreatic duct (hemosuccus pancreaticus) (20.3%), and hematemesis (14.8%) after abdominal pain (29.5%). Around 2.5% of cases present incidentally [1]. The size of the pseudoaneurysm is not a determinant of rupture [4]. The risk of rupture of a splenic artery pseudoaneurysm is 37% with the mortality rate of 90% when untreated [3]. In case of pancreatitis, unexplained anemia or recurrent upper GI bleed should highly raise the suspicion of ruptured psuedoanuerysm or sometimes of a syndrome known as hemosuccus pancreaticus [2].

Aneurysms and pseudoaneurysms have totally different ways to approach for management [5]. Recent data suggest that symptomatic and high risk splenic artery aneurysms should be promptly treated [6]. Patients for surgery should be selected carefully as it is associated with mortality rate of more than 0.5% [7]. Treatment mainly depends upon the location of aneurysm, for proximal and mid aneurysm, resection with proximal and distal ligation of the feeding artery is adequate while for distally located aneurysm resection with splenectomy is the ideal treatment [5].

Nowadays catheter embolisation is also a good option with comparable results [8, 9]. As this pseudoaneurysm is more prone to rupture and carries high mortality rate, the earliest possible intervention is warranted. Splenectomy with or without partial pancreatectomy is now considered the treatment of choice [10]. Surgical intervention carries mortality and morbidity risks of 1.3% and 9%, respectively [10].

## 4. Conclusion

In conclusion, early diagnosis and radical treatment of symptomatic peripancreatic pseudoaneurysms are mandatory. When transcatheter embolisation is unsuccessful and in the absence of acute bleeding that requires emergency surgery, a contrast enhanced computed tomography just before elective surgery may be useful as thrombosis may occur. Further investigations about somatostatin in this field could also be of interest.

## References

[1] Tessier DJ, Stone WM, Fowl RJ (2003). Clinical features and management of splenic artery pseudoaneurysm: case series and cumulative review of literature. *Journal of Vascular Surgery*.

[2] Shah S, Dani S, Shah RV (2002). Pseudoaneurysm from splenic artery associated with chronic pancreatitis. *Indian Journal of Radiology and Imaging*.

[3] Aziz F, Savino JA, Itani MS Pancreatic pseudoaneurysm.

[4] Agrawal GA, Johnson PT, Fishman EK (2007). Splenic artery aneurysms and pseudoaneurysms: clinical distinctions and CT appearances. *American Journal of Roentgenology*.

[5] de Perrot M, Buhler L, Schneider P, Mentha G, Morel P (1999). Do aneurysms and pseudoaneurysms of the splenic artery require different surgical strategy?. *Hepato-Gastroenterology*.

[6] Abbas MA, Stone WM, Fowl RJ (2002). Splenic artery aneurysms: two decades experience at Mayo clinic. *Annals of Vascular Surgery*.

[7] Mattar SG, Lumsden AB (1995). The management of splenic artery aneurysms: experience with 23 cases. *American Journal of Surgery*.

[8] Arepally A, Dagli M, Hofmann LV, Kim HS, Cooper M, Klein A (2002). Treatment of splenic artery aneurysm with use of a stent-graft. *Journal of Vascular and Interventional Radiology*.

[9] Dave SP, Reis ED, Hossain A, Taub PJ, Kerstein MD, Hollier LH (2000). Splenic artery aneurysm in the 1990s. *Annals of Vascular Surgery*.

[10] Guillon R, Garcier JM, Abergel A (2003). Management of splenic artery aneurysms and false aneurysms with endovascular treatment in 12 patients. *CardioVascular and Interventional Radiology*.

